# Structural basis of molecular recognition of helical histone H3 tail by PHD finger domains

**DOI:** 10.1042/BCJ20161053

**Published:** 2017-05-04

**Authors:** Alessio Bortoluzzi, Anastasia Amato, Xavier Lucas, Manuel Blank, Alessio Ciulli

**Affiliations:** Division of Biological Chemistry and Drug Discovery, School of Life Sciences, University of Dundee, James Black Centre, Dow Street, Dundee DD1 5EH, U.K.

**Keywords:** epigenetics, histone binding, PHD finger, protein–protein interactions, reader domains

## Abstract

The plant homeodomain (PHD) fingers are among the largest family of epigenetic domains, first characterized as readers of methylated H3K4. Readout of histone post-translational modifications by PHDs has been the subject of intense investigation; however, less is known about the recognition of secondary structure features within the histone tail itself. We solved the crystal structure of the PHD finger of the bromodomain adjacent to zinc finger 2A [BAZ2A, also known as TIP5 (TTF-I/interacting protein 5)] in complex with unmodified N-terminal histone H3 tail. The peptide is bound in a helical folded-back conformation after K4, induced by an acidic patch on the protein surface that prevents peptide binding in an extended conformation. Structural bioinformatics analyses identify a conserved Asp/Glu residue that we name ‘acidic wall’, found to be mutually exclusive with the conserved Trp for K4Me recognition. Neutralization or inversion of the charges at the acidic wall patch in BAZ2A, and homologous BAZ2B, weakened H3 binding. We identify simple mutations on H3 that strikingly enhance or reduce binding, as a result of their stabilization or destabilization of H3 helicity. Our work unravels the structural basis for binding of the helical H3 tail by PHD fingers and suggests that molecular recognition of secondary structure motifs within histone tails could represent an additional layer of regulation in epigenetic processes.

## Introduction

The plant homeodomain (PHD) finger is one of the largest families of epigenetic reader domains present in chromatin-related proteins, with over 170 PHD fingers identified in the human genome [1]. Early pioneering studies led to PHD fingers being classified as domains that specifically recognize histone H3 trimethylated at K4 [2–5]. However, the diversity of PHD fingers in terms of their ability to recognize a wide array of post-translational modifications (PTMs) and unmodified tails has now become apparent [6–9]. Several PHDs have been characterized that recognize different PTMs on the H3 tail, including di- and tri-methylation of K4 [2], trimethylation of K9 [10], acetylation of K14 [11] and trimethylation of K36 as well as PTMs on H4 such as acetylation [6]. An additional layer of complexity in the molecular recognition by PHD fingers is imparted by the recurrent presence of adjacent domains that aid combinatorial, multivalent readout of histone tails, intra- or inter-nucleosomal [8,12]. Indeed, PHD fingers are often found in close proximity with a bromodomain (BRD) [13,14], as well as other PHD fingers [11,15], bromo-adjacent homology domains [16], tudor domains [17] and chromodomains [18]. Structural studies have elucidated diverse modes of combinatorial readout by PHD fingers and their tandem domains for individual and multiple histone tails, which typically involve recognition of the peptide in a fully extended conformation [9,19]. While PTM-specific and combinatorial readout modalities of histone tails are well understood [20], much less is known about the recognition of secondary structure features within the histone tail itself.

Members of the BAZ family of proteins, which includes BAZ1A, also known as Acf1 [21,22], BAZ1B, also known as Wstf [23,24], BAZ2A, also known as TIP5 (TTF-I/interacting protein 5) [25,26], and BAZ2B [26], are all characterized by the presence of a PHD–BRD tandem module at their C-terminus. BAZ2A is the best characterized member of the BAZ family from a functional standpoint. BAZ2A binds to the ATPase SNF2h (sucrose nonfermenting protein 2 homolog) to form the chromatin remodeling complex NoRC (nucleolar remodeling complex), which plays an essential role in silencing ribosomal DNA (rDNA) genes [27]. Experiments performed with truncated versions of BAZ2A showed that the PHD–BRD module plays an important role in NoRC formation, with the PHD domain being required for interaction with the nucleosome to trigger transcriptional silencing of rDNA [28]. In a recent study, BAZ2A was found to be overexpressed in prostate cancer and a role was proposed for the protein in establishing epigenetic alterations that favor an aggressive phenotype of the cancer [29]. The related protein BAZ2B [30] is yet poorly characterized and its biological role remains unclear. We recently biochemically and structurally characterized the PHD fingers and BRDs of both BAZ2A and BAZ2B, and identified the N-terminal tail of histone H3 as the preferred binding partner of the PHD domains [26]. Structural studies with PHD–BRD tandem constructs have pointed to rather elongated and rigid structures with the two domains probably recognizing distinct regions of H3 histone tails independently [26]*.* NMR spectroscopy has been combined with computational studies to throw light on the molecular recognition features of histone H3K14ac recognition by the BAZ2B BRD [31]. However, the complete molecular picture of H3 tail recognition by the PHD fingers of BAZ2A and BAZ2B had remained elusive.

## Results

### BAZ2A PHD recognizes H3 tails in a helical fold

**Figure 1.**
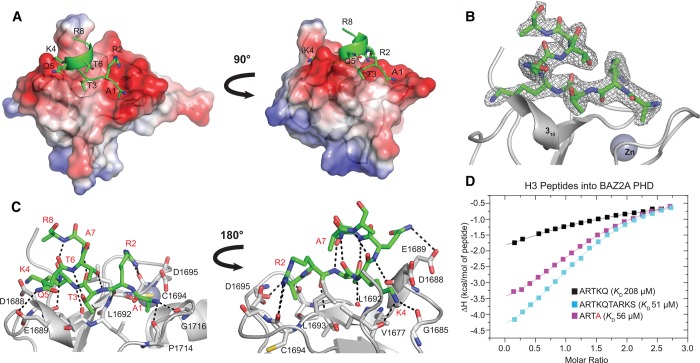
Structural basis of H3 recognition by BAZ2A PHD. (**A**–**C**) Crystal structure of BAZ2A PHD (shown in gray) in complex with H3 10-mer (shown in green). (**A**) Surface and ribbon representation of BAZ2A PHD (regions of positive and negative electrostatic potential are shown in blue and red, respectively) in complex with H3 10-mer shown in a ribbon and stick representation. Residues of the H3 10-mer peptide are labeled. (**B**) The 2*F*_o_–*F*_c_ map contoured at 1*σ* (shown in gray) for H3 10-mer. (**C**) Close-up view of the interaction between the BAZ2A PHD and the H3 10-mer peptide. Residues of BAZ2A PHD interacting with the H3 10-mer peptide are shown in a stick representation and labeled in black. Residues of the H3 10-mer peptide are labeled in red. (**D**) ITC-binding curves of different H3-derived peptides titrated into BAZ2A PHD.

To elucidate the molecular detail of histone H3 N-terminal tail recognition, we solved the crystal structure of ARTKQTARKS (H3 10-mer) bound to BAZ2A PHD (Figure 1A–C; see Table 1 for X-ray data collection and refinement statistics). The peptide residues A1-K4 form an antiparallel β-sheet with the first β-strand of BAZ2A PHD, anchored by backbone hydrogen bonds with residues D1688, L1692, L1693, P1714 and G1716 (Figure 1C). This region of the peptide is found essentially in the same conformation observed in the crystal structure of BAZ2A PHD with bound H3 5-mer (ARTKQ) [26]. The methyl groups of A1 and T3 contribute hydrophobic interactions to peptide binding, and further contributions are brought by the hydrogen bonds and electrostatic interactions of R2 and K4 side chains (Figure 1C). However, starting from K4, the peptide adopts a helical fold that extends at least until R8, forming a complete loop of an α-helix (Figure 1A). The canonical intrapeptide i to i + 4 backbone hydrogen bonds stabilize the helix loop i.e. T3 to A7 and K4 to R8 (Figure 1C). Two additional side chain-to-backbone intramolecular hydrogen bonds are formed, one between the T3 hydroxyl group and the amino group of T6, and a second one between the hydroxyl group of T6 and the amino group of R2 (Figure 1C). Phosphorylation of T3 and methylation of R2 had been shown to lower the binding between BAZ2A PHD and H3 peptide [26], consistent with disruption of these interactions. The electron density for the side chain of R8 is incomplete after Cβ (Figure 1B). There is no interpretable density for K9 and S10, suggesting that these are disordered (Figure 1B). The fold assumed by the peptide is not influenced by crystal contacts. Inspection of the binding pockets in each of the four chains of the asymmetric unit reveals that the histone-binding sites of chains A and D are both occupied by H3 10-mer and are free from crystal contacts that might interfere with or modulate the secondary structure of the peptide itself. Conversely, crystal packing occludes the binding sites of chains B and C and no peptide is found bound to these protomers.

**Table 1:** Crystallographic data processing and refinement statistics Values in parentheses are for the highest resolution shell.

Protein ID	BAZ2A PHD
Ligand	ARTKQTARKS
Beamline	ID29
Synchrotron	ESRF
Processing statistics	
Space group	*P*4_3_2_1_2
Unit cell parameters	
*a*, *b*, *c* (Å)	72.61, 72.61, 99.43
*α*, *β*, *γ* (°)	90.0, 90.0, 90.0
Resolution limits (Å)	45.62–2.4 (2.49–2.4)
Unique observations	10 901 (1132)
Completeness (%)	99.6 (100)
Redundancy	5.4 (5.8)
*R*_merge_ (%)	10.2 (72.8)
*I*/*σI*	9.3 (2.3)
CC_1/2_ (%)	99.4 (71.8)
Wavelength	0.9762
Refinement statistics	
Resolution limits (Å)	58.64–2.4 (2.462–2.4)
*R*_work_/*R*_free_ (%)	18.5 (25.0)/23.5 (29.6)
Number of reflections	10 309 (769)
Number of atoms	1860
Protein/other/solvent	1740/24/96
Average *B* factors (Å)^2^	52.13
RMSD bond (Å)	0.01
RMSD angle (°)	1.50
Ramachandran statistics	
Favored (%)	97.2
Allowed (%)	2.8
Outliers (%)	0.0
PDB code	5T8R

BAZ2A PHD, and its homologous BAZ2B PHD, each binds H3 10-mer with an affinity ∼4-fold higher compared with H3 5-mer (Figure 1D, Supplementary Figure S1 and Table 2). However strikingly, the structure shows no direct interactions between residues T6-S10 and the protein, besides a potential long-range hydrophobic contact between the A7 methyl group and the L1693 side chain. We thus hypothesized that the extra affinity observed with the longer peptide could arise from intramolecular stabilization of its helical fold that helps to avoid clashes with the protein. Indeed, the structure of BAZ2A PHD would be incompatible with a fully extended binding mode of H3 that is commonly observed in PHD-bound crystal structures (Supplementary Figure S3). The 3_10_ helix of BAZ2A PHD blocks H3 from binding in such an extended conformation, forcing it to fold back (Supplementary Figure S3). Consistent with these observations, shorter tetrameric peptides ARTK and ARTA, designed to reduce steric clashes with the 3_10_ helix, bound tighter than H3 5-mer to the BAZ2A PHD domain, and remarkably ARTA bound with comparable affinity to H3 10-mer (Figure 1D, Supplementary Figure S1 and Table 2).

**Table 2:** Summary of thermodynamic-binding parameters for complex formation between different H3 peptides and WT and mutant BAZ2A/B PHD fingers Error values reported on dissociation constant (*K*_D_), stoichiometry of binding (*N*) and binding enthalpy (Δ*H*) are generated by the Origin program and reflect the quality of the fit between the nonlinear least-squares curve and the experimental data. Errors reported on *T*Δ*S* and Δ*G* were propagated from the errors of *K*_D_ and Δ*H*. Raw ITC data are shown for each titration in Supplementary Data (Supplementary Figure S1).

Peptide	Protein	*K*_D_ (µM)	*N*	Δ*H* (kcal/mol)	*T*Δ*S* (kcal/mol)	Δ*G* (kcal/mol)
ARTK	BAZ2A PHD	100 ± 4	1.57 ± 0.03	−6.1 ± 0.2	−0.6 ± 0.2	−5.46 ± 0.02
ARTA	BAZ2A PHD	56 ± 2	1.44 ± 0.01	−4.7 ± 0.1	1.1 ± 0.1	−5.81 ± 0.02
ARTKQ	BAZ2A PHD	210 ± 30	1.6 ± 0.1	−3.7 ± 0.4	1.3 ± 0.4	−5.0 ± 0.1
ARTKQTARKS	BAZ2A PHD	51 ± 2	1.35 ± 0.01	−5.8 ± 0.1	0.1 ± 0.1	−5.86 ± 0.02
AATKQTARKS	BAZ2A PHD	>1000	N.D.	N.D.	N.D.	N.D.
ARAKQTARKS	BAZ2A PHD	No binding				
ARTAQTARKS	BAZ2A PHD	42 ± 2	1.03 ± 0.02	−6.1 ± 0.2	−0.1 ± 0.2	−6.00 ± 0.03
ARTKATARKS	BAZ2A PHD	9.3 ± 0.2	1.21 ± 0.01	−6.13 ± 0.02	0.74 ± 0.02	−6.87 ± 0.01
ARTKQAARKS	BAZ2A PHD	55 ± 2	0.90 ± 0.01	−5.6 ± 0.1	0.2 ± 0.1	−5.82 ± 0.02
ARTAATARKS	BAZ2A PHD	12.1 ± 0.3	1.32 ± 0.01	−7.01 ± 0.04	−0.30 ± 0.04	−6.71 ± 0.02
ARTGGTARKS	BAZ2A PHD	143 ± 7	1.28 ± 0.04	−7.4 ± 0.3	−2.2 ± 0.3	−5.25 ± 0.03
ARTKQ	BAZ2B PHD	190 ± 25	1.0 ± 0.1	−4.7 ± 0.8	0.4 ± 0.8	−5.1 ± 0.1
ARTKQTARKS	BAZ2B PHD	40.0 ± 0.5	1.45 ± 0.04	−5.43 ± 0.02	0.58 ± 0.02	−6.01 ± 0.01
AATKQTARKS	BAZ2B PHD	>1000	N.D.	N.D.	N.D.	N.D.
ARAKQTARKS	BAZ2B PHD	>5000	N.D.	N.D.	N.D.	N.D.
ARTAQTARKS	BAZ2B PHD	8.5 ± 0.2	0.88 ± 0.01	−6.62 ± 0.04	0.30 ± 0.05	−6.92 ± 0.01
ARTKATARKS	BAZ2B PHD	7.0 ± 0.2	1.20 ± 0.03	−6.18 ± 0.02	0.86 ± 0.03	−7.04 ± 0.02
ARTKQAARKS	BAZ2B PHD	44.6 ± 0.8	1.01 ± 0.01	−5.3 ± 0.1	0.68 ± 0.06	−5.94 ± 0.01
ARTAATARKS	BAZ2B PHD	2.6 ± 0.1	1.13 ± 0.01	−7.12 ± 0.02	0.50 ± 0.03	−7.63 ± 0.02
ARTGGTARKS	BAZ2B PHD	166 ± 8	1.3 ± 0.1	−8.4 ± 0.5	−3.3 ± 0.5	−5.16 ± 0.03

Abbreviations: N.D.: not determined.

### Structural basis of recognition of helical H3 N-terminal tail by PHD fingers

**Figure 2.**
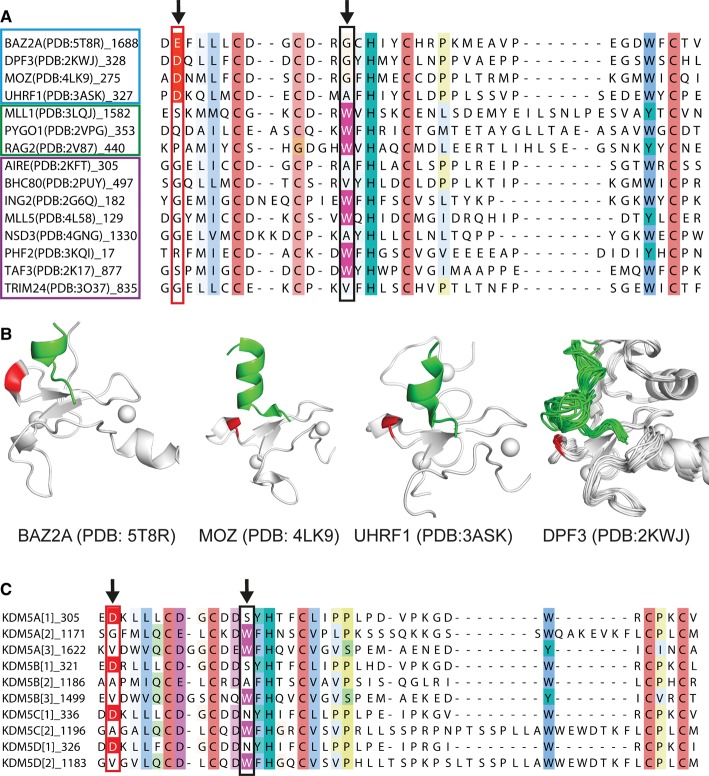
‘Acidic wall’ residue is conserved among PHD fingers that recognize helical H3 tail. (**A**) Sequence alignment of PHD fingers whose structure was solved in complex with an H3 N-terminal tail peptide. The column corresponding to E1689 of BAZ2A is highlighted through the alignment with a red box, and Asp or Glu residues in this column are colored in red. The column corresponding to the absolutely conserved tryptophan of PHD fingers that recognize methylated-K4 is highlighted through the alignment with a black box, and tryptophan residues in this column are colored in magenta. PHD fingers that induce the H3 tail to adopt a helical (cyan box), bent (green box) or extended (magenta box) fold are grouped (see Supplementary Figure S4 for full alignment). (**B**) Structures of PHD fingers (gray cartoon) that have an acidic residue (shown in red) in the position corresponding to E1689 of BAZ2A and that induce the H3 N-terminal peptide (green cartoon) to adopt a helical folded-back conformation. (**C**) Sequence alignment of PHD fingers of KDM5 proteins. The columns corresponding to E1689 of BAZ2A and to the absolutely conserved tryptophan of PHD fingers that recognize methylated-K4 are highlighted through the alignment as described in **A** (see Supplementary Figure S5 for full alignment).

Our structural and biophysical data point to an important role of the H3 tail helicity in the recognition of PHD fingers. To assess the prevalence of this recognition mode, we inspected all structures of PHD fingers in complex with H3 peptides deposited in the Protein Data Bank (PDB). Our analysis revealed that the conformation assumed by residues A1-K4 of H3 upon binding to a PHD finger is normally extended and relatively well conserved. In contrast, the folding of the peptide from K4 onwards varies from a completely extended conformation, e.g. H3 N-terminal peptide bound to the PHD domain of ING2 (PDB: 2G6Q [5]), to an α-helix, e.g. H3 N-terminal peptide bound to the double PHD finger (DPF) of MOZ (PDB: 4LK9 [15]). We identified three possible conformations that an H3 N-terminal peptide can adopt when bound to a PHD finger: helical, bent and fully extended (Figure 2A and Supplementary Figure S4). Interestingly, H3 assumes a helical fold when in complex with PHD fingers that harbor a short helical turn or loop just before the first β-strand. This is a 3_10_ helix in the case of BAZ2A PHD (Figure 1). We noted that the 3_10_ helix is particularly acidic in BAZ2A PHD and in its close homolog BAZ2B PHD, comprising D1688 and E1689 in BAZ2A and E1943 and E1944 in BAZ2B. To investigate the conservation of this structural feature, we performed a multiple sequence alignment with PHD fingers whose structure was solved in complex with an H3 N-terminal peptide (Figure 2A and see Supplementary Figure S4A for full alignment). We observed that the PHD fingers of BAZ2A, UHRF1, MOZ and DPF3 all have a conserved acidic residue in the position corresponding to E1689 of BAZ2A PHD, and all recognize H3 in a folded-back helical conformation starting from K4 onwards (Figure 2B). This acidic residue, which we name ‘acidic wall’, is also structurally conserved, as in all cases it is positioned against the bottom of the first loop formed by H3 (Figure 2B). Topological conservation suggests that the negatively charged carboxylate may help to stabilize the positive dipole of the N-terminus of the helix [32]. In contrast with the recognition of the helical H3 tail, the bent conformation of H3 bound to the PHD appears to be stabilized by a different set of interactions (Supplementary Figure S4C).

### Prevalence of helical H3 N-terminal tail-recognizing human PHD fingers

It is remarkable that PHD fingers recognizing H3 in a bent or extended conformation do not have an acidic residue in the position corresponding to E1689 of BAZ2A PHD (Figure 2A and Supplementary Figure S4). To investigate the prevalence of the acidic wall residues in all human PHD fingers, we extended our bioinformatics analysis to the entire human genome [1]. We found that 36 of the 172 sequences annotated as PHD fingers have an acidic residue in the position that corresponds to E1689 of BAZ2A (Supplementary Figure S5). Among these are all the PHD fingers of the BAZ family, CREBBP [33] and the homologous EP300 [34], all members of the DPF family of proteins: DPF1, DPF2 and DPF3, as well as members of the KDM5/JARID1 histone lysine demethylase family: KDM5A, KDM5B, KDM5C and KDM5D [35,36]. Interestingly, we noted that, in all four KDM5 members, only the first PHD domain, which like BAZ2A/B recognizes unmodified K4, but not the second or third, has an acidic residue at this position, and this is mutually exclusive with the presence of the conserved tryptophan residue characteristic of the aromatic cage for methyl-K4 recognition (Figure 2C) [37]. Indeed, only 5 of the 36 sequences containing the acidic wall residue also contain this tryptophan (Supplementary Figure S5). Based on this observation, we postulate that there could be a level of incompatibility between methyl-lysine readout and helical H3 recognition by PHD finger domains. Five PHD fingers bear both an acidic wall residue and the tryptophan needed for methyl-K4 recognition: ASH2L [38], the MLL2 and MLL3 members of the KMT2 family of lysine methyltransferases [39], PHF20 [40] and UBR7 (Supplementary Figure S5). Structural information is available only for ASH2L PHD, which unveils an atypical PHD fold with only one zinc ion coordinated, suggesting that the ASH2L PHD structure is incompatible with histone binding [38]. The remaining four PHD fingers are poorly characterized, their substrate specificity is not known and it is difficult to conclude if they represent genuine exceptions to the observed mutual exclusivity between acidic wall residue and conserved Trp residue.

### Characterization of the interaction between BAZ2A and BAZ2B PHD with H3 N-terminal tail by NMR

**Figure 3.**
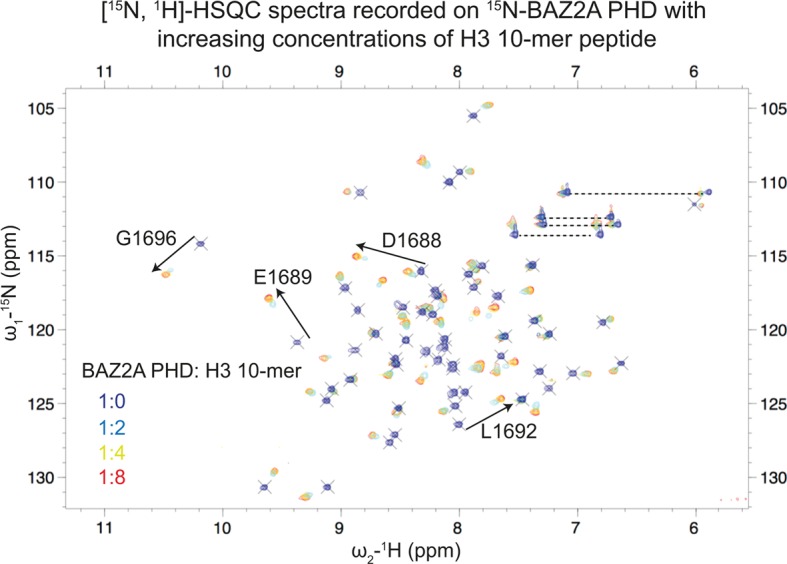
CSPs induced by the H3 10-mer peptide on BAZ2A PHD. Overlay of [^15^N-^1^H]-HSQC spectra recorded on ^15^N-BAZ2A PHD with increasing concentrations of the H3 10-mer peptide. Spectra were recorded at the following protein:peptide molar ratios: 1:0 (blue), 1:2 (cyan), 1:4 (yellow) and 1:8 (red). For a set of peaks, the direction of the shifts is indicated with black arrows. The horizontal dotted lines indicate peak pairs corresponding to the side-chain of Asn and Gln.

**Figure 4.**
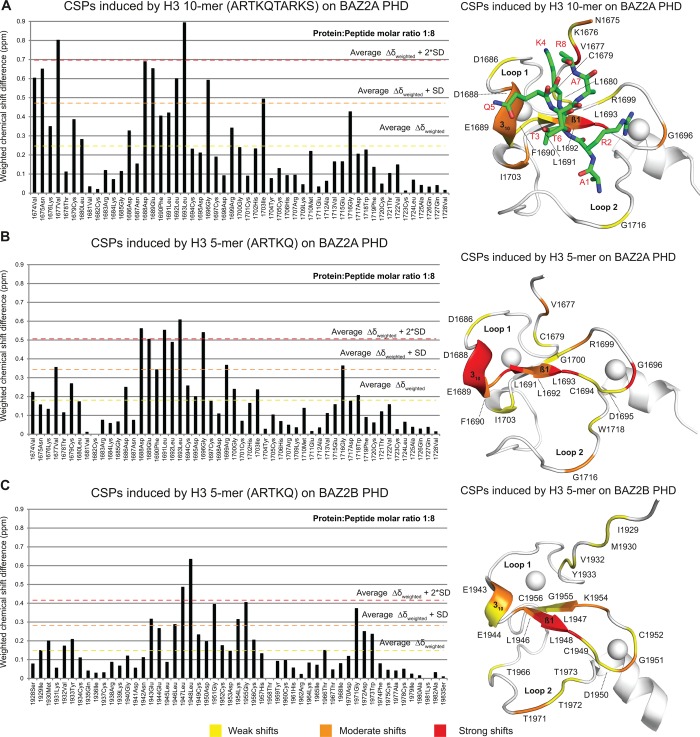
Characterization of the interaction between H3 N-terminal tail and BAZ2A/B PHD in solution by NMR. Chemical shift differences induced by H3-derived peptides on BAZ2A/B PHDs were weighted as described in the Experimental section and plotted against BAZ2A/B PHD sequences. The resulting histograms were used to group residues based on the extent of their CSPs: weak (weighted chemical shift difference value equal or above the average chemical shift), medium (equal or above the average chemical shift plus the standard deviation) and strong (equal or above the average chemical shift plus two times the standard deviation). The CSPs observed were mapped on BAZ2A/B PHDs structures (PDB: 5T8R and 4QF3, respectively) by coloring residues with weak shifts in yellow, medium in orange and strong in red. Residues with a weighted chemical shift difference value lower than the average chemical shift are in white. The H3 10-mer peptide is shown as sticks and colored in green and its residues are labeled in red. In the middle panel, the peptide is omitted for clarity.

The small PHDs (∼6.5 kDa) of both BAZ2A and BAZ2B yielded high-quality [^15^N-^1^H] heteronuclear single-quantum coherence (HSQC) spectra (Figure 3 and Supplementary Figure S6), and all the backbone amide protons were readily assigned in both constructs except for the first serine residue of BAZ2A PHD [Biological Magnetic Resonance Bank (BMRB) deposition nos **26** **754** and **25** **988**, for BAZ2A PHD and BAZ2B PHD, respectively]. First, we validated our co-crystal structure of BAZ2A PHD in complex with H3 10-mer. [^15^N-^1^H]-HSQC spectra were recorded on a sample of ^15^N-labeled BAZ2A PHD at increasing concentrations of H3 10-mer (Figure 3). The shifts observed were quantified and mapped on to the structure of the BAZ2A PHD-H3 10-mer complex (Figure 4A). Strong and moderate shifts were found to cluster at β1 and at the 3_10_ helix, with the acidic patch residues D1688 and E1689 giving some of the strongest shifts (Figures 3 and 4A). Additional shifts that recapitulate the contacts observed in the crystal structure include G1716, whose carbonyl group engages in a hydrogen bond with the H3 N-terminus and at the protein N-terminus close to the K4 side chain (Figure 4A). Other shifts are observed for residues of BAZ2A PHD relatively far from the H3 10-mer binding site, e.g. G1696, on a loop that links β1 and β2 strands, and I1703, on a short helix after β2 (Figure 4A). Next, we applied the same procedure to yield a chemical shift perturbation (CSP) histogram and corresponding heat map representative of the binding site of the shorter H3 5-mer (Figure 4B). The chemical shift changes induced by H3 5-mer and H3 10-mer closely overlap, showing minor differences only at the N-terminus of BAZ2A PHD where the H3 10-mer induces additional shifts compared with H3 5-mer (Figure 4A,B). Importantly, we did not observe extra shift clusters that could suggest the presence of additional binding sites exploited by the longer H3 10-mer, consistent with the binding mode observed in our crystal structure. Finally, we studied the binding of the H3 N-terminal tail to BAZ2B PHD by NMR. As in BAZ2A PHD, BAZ2B PHD also harbors an acidic wall residue, E1944, and a 3_10_ helix positioned just before β1 (Figure 4). We found that the chemical shift changes induced by H3 5-mer on BAZ2B PHD (Figure 4C and Supplementary Figure S6B) are remarkably consistent with the ones observed for BAZ2A PHD and most shifts map at equivalent positions in the two PHD fingers (Figure 4), including the acidic wall. Overall, the NMR data suggest a probably conserved molecular recognition of the H3 N-terminal tail by the homologous BAZ2A/B PHD fingers (sequence identity of 66%).

### Role of the acidic wall residue of BAZ2A and BAZ2B PHD fingers in H3 N-terminal tail recognition

**Figure 5.**
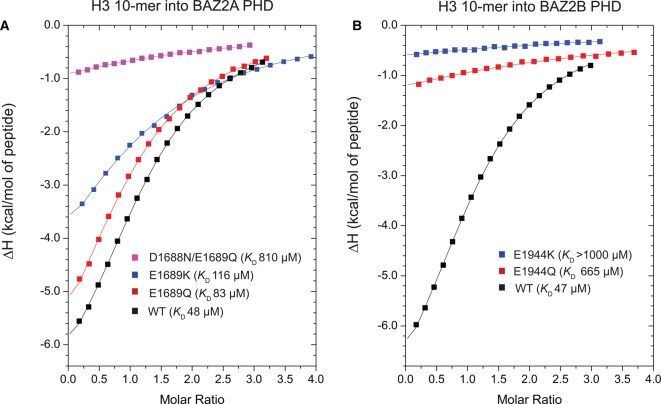
Role of the acidic wall residue of BAZ2A/B PHDs in H3 N-terminal tail recognition. (**A** and **B**) ITC-binding curves of the H3 10-mer peptide titrated into WT and mutant BAZ2A PHD (**A**) and BAZ2B PHD (**B**).

To investigate the role of the acidic patch in H3 N-terminal tail recognition, we mutated E1689 of BAZ2A PHD to Gln and Lys, aiming to neutralize and invert, respectively, the negative charge of the acidic wall side chain. Equivalent mutations were also introduced at the acidic wall of BAZ2B PHD, namely E1944Q and E1944K. Correct folding of the resulting mutants was confirmed by ^1^H 1D NMR spectra (Supplementary Figure S7). Mutant proteins were compared with wild type (WT) for their ability to bind H3 10-mer peptide by isothermal titration calorimetry (ITC; Figure 5, Supplementary Figure S2 and Table 3). Mutation of the acidic wall to Gln led to a decrease in binding affinity, up to 8-fold with BAZ2B PHD. The effect was even more pronounced when the charge was inverted, as the E1944K mutation completely abrogated binding (Table 3 and Figure 5B). Mutation of the acidic wall residue in BAZ2A PHD also affected the thermodynamic parameters of H3 binding, albeit more moderately than for BAZ2B (Figure 5). Specifically, the E1689Q mutation weakened the binding affinity by ∼2-fold, whereas the E1689K mutant showed a loss of binding affinity of 2.4-fold (Table 3 and Figure 5A). We noted that in BAZ2A PHD, the residue just preceding E1689 is also acidic (D1688), and in our crystal structure, its side chain forms one side of the pocket that accommodates the K4 side chain of the H3 peptide (Figure 1C). Superposition with other PHD structures bound to the helical H3 tail suggested that the R8 side chain of H3 points backward toward the acidic patch of BAZ2A, and could form a salt bridge with the carboxylate group of the D1688 side chain (Figure 1C). Moreover, in the NMR HSQC spectra, the amide NH of D1688 exhibited large chemical shifts in the presence of the H3 peptide (Figure 3). Based on these observations, we hypothesized that this residue could also be important for binding and could potentially compensate the E1689Q mutation. We therefore designed and expressed a double mutant D1688N/E1689Q to fully neutralize the negative charges on the 3_10_ helix of the acidic wall. This double mutation drastically affected the binding of BAZ2A PHD toward the cognate H3 histone peptide, reducing the binding affinity by 17-fold (Figure 5A and Supplementary Figure S2). Taken together, these data demonstrate that the negatively charged patch corresponding to the acidic wall is an important feature for the recognition of the H3 N-terminal tail by the PHD fingers of BAZ2A and BAZ2B.

**Table 3:** Summary of the thermodynamic-binding parameters for complex formation between H3 WT 10-mer peptide and mutant BAZ2A/B PHD Titrations were performed at 25°C in triplicate, except where indicated, and values reported are the means ± s.e.m. Raw ITC data are shown for representative titrations in Supplementary Data (Supplementary Figure S2).

Protein	*K*_D_ (µM)	*N*	Δ*H* (kcal/mol)	*T*Δ*S* (kcal/mol)	Δ*G* (kcal/mol)
BAZ2A PHD wt	48 ± 2	1.29 ± 0.01	−7.9 ± 0.1	−2.0 ± 0.1	−5.90 ± 0.02
BAZ2A PHD E1689Q	83 ± 7	1.02 ± 0.03	−9.6 ± 0.7	−4.0 ± 0.8	−5.57 ± 0.05
BAZ2A PHD E1689K*	116 ± 6	1.1 ± 0.4	−7.6 ± 2.5	−2.2 ± 0.4	−5.37 ± 0.03
BAZ2A PHD D1688N/E1689Q	810 ± 50	1^†^	−9.0 ± 1.2	−4.8 ± 1.2	−4.22 ± 0.04
BAZ2B PHD wt	47 ± 2	1.17 ± 0.02	−8.3 ± 0.2	−2.4 ± 0.2	−5.90 ± 0.03
BAZ2B PHD E1944Q	660 ± 60	1^†^	−8.5 ± 1.1	−4.1 ± 1.1	−4.34 ± 0.06
BAZ2B PHD E1944K	>1000	N.D.	N.D.	N.D.	N.D.

* Titrations performed in duplicate.

† *N* was fixed to 1 during the data fitting.

### Changes in H3 N-terminal tail helicity correlate with different binding affinities for BAZ2A and BAZ2B PHD fingers

To gain a better understanding of the histone molecular recognition, we investigated the energetic contribution of different H3 residues in binding to the PHD fingers of BAZ2A and BAZ2B. We performed an alanine scan where residues 2–6 of the H3 10-mer were mutated individually to alanine and the resulting mutant peptides tested for binding with BAZ2A PHD and BAZ2B PHD by ITC (Supplementary Figure S1 and Table 2). The R2A and T3A mutations abolished binding. The K4A mutation did not affect the binding affinity with BAZ2A PHD and even increased the affinity toward BAZ2B PHD. The Q5A mutation improved binding, and the simultaneous introduction of K4A and Q5A mutations remarkably increased binding affinities by 4-fold (BAZ2A) and 15-fold (BAZ2B) (Table 2). Finally, T6A did not affect the binding affinity of H3 10-mer toward either protein. Our data show that K4-T6 residues are not critical for binding to the PHD fingers of BAZ2A and BAZ2B, while R2-T3 are crucial. These results are consistent with those recently reported by Chakravarty et al. [37] for BAZ2A PHD and the first PHD domain of KDM5B but are distinct from the results of the first PHD of AIRE, which is known to bind H3 in an extended conformation. In that case, the T3A mutation was tolerated, whereas the K4A mutation abolished binding [37].

The increase in binding affinity observed for the mutant H3 peptides was unexpected, especially the ones harboring the K4A mutation as both BAZ2A and BAZ2B PHD fingers recognize unmodified K4 [26]. The strong contacts formed by the K4 side chain in the deep surface groove of the PHD surface (Figure 1A–C) would not be recapitulated upon K4A mutation, and hence, a loss of binding affinity was anticipated. To investigate the structural basis for the unusual increase in binding affinity of the H3 10-mer AA mutant peptide (ARTAATARKS), we mapped its binding site by NMR using the so-called minimal shift approach (Supplementary Figure S8). Overall, we observed equivalent CSP maps for the H3 10-mer AA mutant compared with WT peptide (Supplementary Figure S8), the major difference being present at the N-terminus of BAZ2A PHD where the side chain of H3K4 is accommodated. Consistently with the H3 K4A mutation, the shifts induced by the H3 10-mer WT peptide at the BAZ2A PHD N-terminus are reduced for the AA mutant peptide (Supplementary Figure S8). Importantly, we did not observe any extra cluster of shifts for H3 10-mer AA mutant peptide that would suggest different binding site(s) exploited by this mutant peptide (Supplementary Figure S8). In light of our crystal structure and of the helical fold of bound H3 peptide, we reasoned that the K4A and Q5A mutations could stabilize the peptide helicity accounting for the increased affinity. Indeed, alanine has the highest helix propensity among natural amino acids [41].

**Figure 6.**
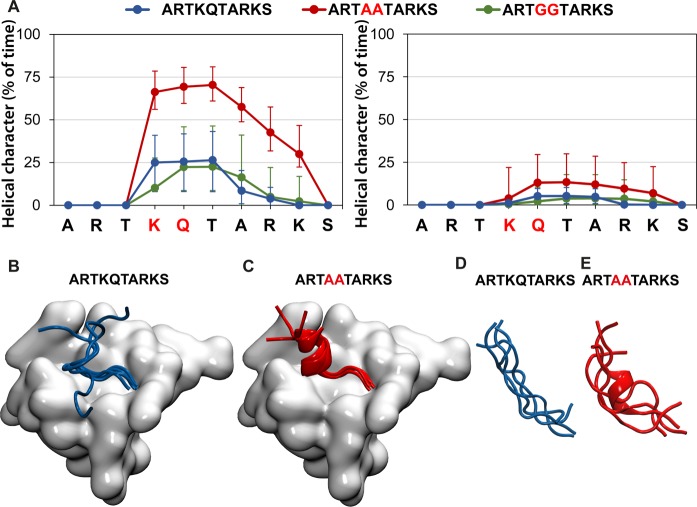
MD simulations of helicity of H3 N-terminal tail, WT and mutant. (**A**) Helical character of each peptide residue in the WT, double-Ala and double-Gly mutants during the last 60 ns of MD simulations, represented as a percentage of time with the secondary structure of α-, 3_10_- or π-helix, and shown as median ± interquartile range, in complex with BAZ2A PHD (left) and in aqueous solution (right). (**B** and **C**) A superposed cartoon representation of the last frame of 4 MD replicas of H3 10-mer (**B**) and H3 10-mer K4A/Q5A (**C**) in complex with BAZ2A PHD (shown as a surface in gray). (**D** and **E**) Superposed cartoon representation of the last frame of four MD replicas of H3 10-mer (**D**) and H3 10-mer K4A/Q5A (**E**) in aqueous solution.

To test this hypothesis, the role of the K4A and Q5A mutations in the helical propensity and stability of H3 10-mer was studied by molecular dynamics (MD) simulations (Figure 6). First, we modeled H3 10-mer in the context of the complex with BAZ2A PHD (Figure 6A, left panel, and B,C). The helical character of each amino acid during the last 60 ns of simulation, reported as a percentage of time with secondary structure of α-, 3_10_- or π-helix, is shown in Figure 6A (in blue). Residues K4-T6 are stabilized ∼25% of the time as a helical structure. The tendency decreases rapidly after T6. A superposition of the last frame of each replica of the simulation shows that, upon unfolding, the C-terminus of H3 10-mer is naturally flexible and disordered (Figure 6B), in agreement with the lack of electron density observed at residues 9 and 10 in the crystal structure (Figure 1B). An analysis of the intramolecular hydrogen-bond contacts occurring within the peptide during the simulation shows that the T3–T6 contact observed in the crystal structure is persistent and well conserved (Supplementary Figure S9). Remarkably, introducing alanine residues at positions 4 and 5 to generate the H3 10-mer AA peptide induces a significant stabilization of the helix along the simulation (*P* < 0.002), which is present over 60% of the time for residues K4-A7 and still over 25% beyond and up to K9 (Figure 6A, left panel, red). The intramolecular hydrogen-bond contacts are consequently strengthened during the simulation and involve residues beyond T6 forming a clear ‘i to i + 4’ pattern characteristic of the α-helix (Supplementary Figure S9).

We hypothesized that the observed increase in helical stability upon alanine mutation could also be reflected in their unbound state. To analyze this effect, we modeled both peptides in aqueous solution (Figure 6A, right panel, and D,E). In the absence of the PHD protein, there is still some helicity, albeit weak, persisting in the WT peptide (∼5% of the time). The helical character of the peptide in the unbound state was consistently increased by the introduction of K4A and Q5A mutations to 12% of the time (Figure 6A, right panel). To further investigate the relationship between the helical propensity of H3 10-mer and binding affinity toward BAZ2A/B PHDs, we aimed to reduce the peptide helicity by replacing K4 and Q5 with a Gly residue. Indeed, excluding proline, glycine has the lowest helix propensity among natural amino acids [41]. The resulting H3 10-mer GG mutant peptide (ARTGGTARKS) showed markedly reduced binding affinity toward both BAZ2A/B PHD fingers (Supplementary Figure S1 and Table 2). ITC data revealed that the decreases in binding affinity of the H3 10-mer GG mutant are contributed entirely by large entropic penalties (Table 2), consistent with a significant reduction in conformational freedom of the peptide upon binding relative to H3 10-mer WT or H3 10-mer AA mutant peptides. MD simulations further showed a significant weakening of the intramolecular hydrogen-bond network along with a modest decrease in the helical character in the H3 10-mer GG peptide compared with H3 10-mer WT and AA mutant (Figure 6A and Supplementary Figures S9 and S10). Taken together, our data reveals that H3 tail helicity plays an important role in recognition by the PHD domains of BAZ2A/B.

### Circular dichroism confirms helicity of H3 N-terminal tail in solution

**Figure 7.**
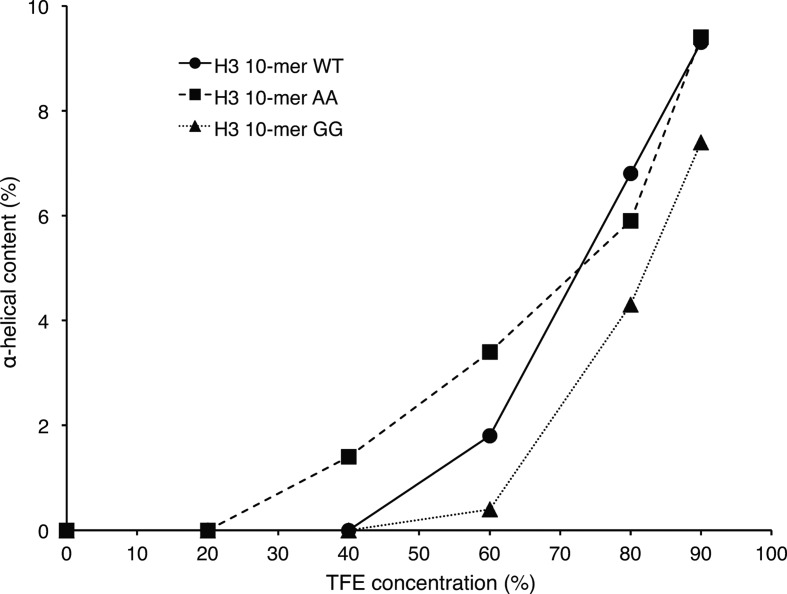
Helical propensity of H3-derived peptides studied by CD. The CD spectra of the H3 10-mer WT (ARTKQTARKS), H3 10-mer AA (ARTAATARKS) and H3 10-mer GG (ARTGGTARKS) peptides at different TFE concentrations (Supplementary Figure S11) were deconvoluted (Supplementary Tables S1–S3) and the content of the regular α-helix found in the best matching solution was plotted against the TFE concentration (v/v).

The helical content of H3 10-mer WT, H3 10-mer AA and H3 10-mer GG peptides in solution was investigated by circular dichroism (CD). The CD spectra recorded in water displayed a depth between 195 and 200 nm (Supplementary Figure S11, top panel), which is a characteristic of disordered proteins or peptides [42]. This is in agreement with the MD results where all three peptides rapidly lost the helical fold used as starting conformation and showed only a modest helical content along the simulation (Figure 6A, right panel). To investigate the propensity of the three peptides to adopt an α-helix fold, CD spectra were recorded at increasing concentrations of 2,2,2-trifluoroethanol (TFE; Supplementary Figure S11). TFE is known to stabilize the helical fold of peptides and is often used to assess their helical propensity [43–46]. The CD spectra of the three peptides obtained at different TFE concentrations were deconvoluted (Supplementary Tables S1–S3) and the α-helix content found was plotted against TFE concentration (Figure 7). During the TFE titration, the α-helix content increases from 0% to ∼10% for all three peptides but with different trends. Indeed, between 40 and 60% TFE, the H3 10-mer AA peptide has the highest α-helix content, followed by H3 10-mer WT and then H3 10-mer GG. The trends observed are in agreement with the expectation that the K4A and Q5A mutations would increase helical propensity of the H3 10-mer peptide and K4G and Q5G reduce it.

## Discussion

Epigenetic regulatory processes modulate human physiology and disease; thus, reaching a comprehensive understanding of their molecular basis is important. Molecular recognition of secondary structural features within histone tails by epigenetic reader domains has received little attention to date. Herein, we have examined the structural and biophysical basis for the recognition of the helical histone H3 tail by PHD fingers, using the PHDs of BAZ2A and BAZ2B as the model system.

Our structural insights into the molecular recognition of histone H3 by the BAZ2A and BAZ2B PHD fingers add to the emerging evidence for specific recognition of the helical H3 tail by this reader family, provided by peptide-bound structures recently solved for the PHD fingers of UHRF1, MOZ and DPF3 (Figure 2B). Identification of a strict conservation for an Asp/Glu residue at the acidic wall position on these family members suggests a simple consensus signature for this subclass of PHD domains. Sequence alignment of the whole human PHD finger-ome identified a single putative exception to this rule, namely the first PHD finger of KDM5B (KDM5B-PHD1) that bears an Asp as the acidic wall residue, in spite of being found to bind H3 in an extended conformation based on an NMR structure of the complex (PDB: 2MNZ [47], Supplementary Figures S4A and S12). However, in that structure, the H3 peptide fits into a groove close to the N-terminus of KDM5B-PHD1 rather than running parallel to the domain as observed for the PHD fingers that bind H3 peptide in an extended conformation (Supplementary Figure S12). Such arrangement resembles the conformation observed for H3 bound to UHRF1 PHD in the co-crystal structure reported by Wang et al. [48] (Supplementary Figure S12). However, there are six other independent structures of UHRF1 PHD in complex with H3 N-terminal peptide bound in a helical fold (Supplementary Figure S12) [17,49–53]. We therefore propose that, as observed for UHRF1, the KDM5B-PHD1 can also recognize the H3 N-terminal tail in a helical fold.

We identify a subclass of 36 human PHD fingers containing an acidic wall residue Asp/Glu as potential consensus to molecular recognition of the helical H3 tail. The minimal overlap observed between the acidic wall subclass and the subclass comprising the key conserved Trp residue corresponding to specific readout of methylated-K4 suggests a level of incompatibility between these two molecular recognition features. Indeed, methylation of H3K4 was found to weaken or completely abrogate histone binding in several PHD fingers that recognize helical H3, such as BAZ2A/B [26], DPF3b [11] and MOZ [54]. This trend of incompatibility is particularly evident in the KDM5 subfamily, where, in all its members, only the first PHD finger (PHD1) has an acidic wall and this is mutually exclusive with the conserved Trp for methylated-K4 recognition that is instead present in PHD2 and PHD3 (Figure 2C). The interaction between unmodified H3 N-terminal peptide and both KDM5A-PHD1 and KDM5B-PHD1 has been recently characterized using NMR [35,36,47]. Interestingly, the patterns of CSPs observed were in both cases consistent with the one observed for BAZ2A/B PHDs (Figure 4).

The region corresponding to the acidic wall residue is often found as highly acidic, with additional Asp/Glu residues found either immediately before or after the acidic wall residue (Figure 2A–C and Supplementary Figure S5). Sequence analysis showed that 22 of these 36 PHD sequences contain at least two adjacent acidic residues (Supplementary Figure S5), suggesting the prevalence of a double acidic patch. We provide evidence that full neutralization of this double acidic patch abrogates H3 binding in BAZ2A PHD, highlighting its important role (Figure 5 and Supplementary Figure S2). We propose that the negatively charged patch at the acidic wall helps to stabilize the helical fold of H3 by forming electrostatic interactions with the positive dipole of the histone helix. The carboxylate side chain(s) at the acidic wall can help to induce the helical bound conformation in H3 by interacting with the basic side chains of K4 and Q5 at the start of the helix. In addition, it can form a salt bridge with the guanidinium group of R8, as shown by recently solved crystal structures of the DPF of DPF3b (PDB: 5I3L [55]), MOZ (PDB: 5B75 [56]) and MORF (PDB: 5U2J [57]), and thus probably occurring in BAZ2A PHD. Interestingly, mutation A275D in the DPF of MOZ just upstream of acidic wall residue D276, thus installing a double-negative charge as in BAZ2A, enhanced the binding of H3K14ac peptide by 3- to 4-fold [54].

It has been shown that PTMs can affect the secondary structure of histones [58]. It is tempting to speculate that induction or stabilization of the helicity of histone tails could represent an additional layer of regulation in epigenetic processes beyond or in cross-talk with PTMs. Within the context of tandem epigenetic reader domains, the H3 helical fold has been shown to be important for simultaneous recognition of distinct regions of the H3 tail by two epigenetic reader domains on the same protein. For example, the H3 helical conformation induced by UHRF1 PHD binding was found to be essential for productive recognition of K9 modification states by the neighboring tudor domain [49]. Similarly, the H3 helical fold was found to be critical for simultaneous recognition of K4 and K14 modifications by the double PHD finger domain of MOZ [15,54,56]. Our work suggests that, in BAZ2A/B and related proteins, the helical fold of the bound H3 N-terminal tail could facilitate productive simultaneous recognition of both unmodified K4 and downstream marks, e.g. K14ac, by the neighboring PHD finger and BRD, respectively, which warrant future investigation.

In conclusion, we propose that among the large PHD family exists a class of PHD fingers with a distinct recognition mode of the histone H3 tail that induces H3 to adopt a helical fold after K4. PHD fingers that belong to this class are characterized by the presence of a conserved Asp/Glu residue within a short acidic patch made of a helical turn or loop just before the first β-strand. We show that H3 helicity is critical for molecular recognition by this subclass of PHD fingers and identify mutations at K4 and Q5 in H3 that either enhance or weaken the binding affinity by stabilizing or disrupting the peptide helicity, respectively. This mutagenesis approach may provide a rapid and direct strategy to identify other reader domains that also recognize helical H3 tails. Our work has also implications for drug design. The growing interest in targeting epigenetic reader domains with small molecules has led to many examples of successful campaigns delivering potent chemical probes in particular for BRDs [59,60], but also for methyl-lysine reader domains such as malignant brain tumor domains [61–63] and chromodomains [63–66]. In contrast, relatively little progress has been made on targeting PHD fingers, with only two examples reporting weak-binding fragments [67] and screening-active compounds [68], suggesting low ligandability for this class of reader domains. Drug design approaches to stabilize the helical conformation, e.g. by using stapled peptides, or to mimic the helix recognition pharmacophore could provide attractive new strategies to aid the development of epigenetic chemical probes that disrupt this class of reader–histone interactions.

## Experimental

### Protein expression and purification

Expression and purification of BAZ2A PHD and BAZ2B PHD were performed as described recently [26]. ^15^N and ^15^N/^13^C uniformly labeled BAZ2A PHD and BAZ2B PHD were expressed in modified M9 minimal medium [69] where the sole sources of nitrogen and carbon were 1 g/l ^15^NH_4_Cl (Goss Scientific) and 2 g/l ^13^C-d-glucose (Goss Scientific) as appropriate. The expression conditions and the purification procedures used for labeled proteins were the same as for unlabeled samples [26].

### Site-directed mutagenesis

Mutations were introduced into BAZ2A/B PHD fingers by polymerase chain reaction (PCR) amplification of the original construct using Phusion DNA Polymerase (Thermo Fisher Scientific) and a specific pair of primers for each mutation (Supplementary Table S4). The PCR amplification product was incubated with Dpn I (New England BioLabs) for 1 h at 37°C to digest the parental DNA strands and then used to transform *Escherichia coli* DH5α cells. Transformed cells were grown on lysogeny broth (LB) agar plates supplemented with 100 µg/ml ampicillin for 16 h at 37°C. Single colonies were picked to inoculate 5 ml of LB plus 100 µg/ml ampicillin and grown for 16 h at 37°C. The DNA was extracted from the bacterial cultures using the QIAprep Spin Miniprep Kit (Qiagen) and the presence of the desired mutation was checked by DNA sequencing. Electrospray ionization mass spectrometry analyses confirmed that the mutant constructs were successfully translated into the correctly mutated proteins.

### Peptide synthesis

Peptides were synthetized by standard automated solid-phase synthesis on a ResPep SL peptide synthesizer (Intavis) using Fmoc-protected amino acids and Rink Amide resin (Novabiochem). Amino acids were coupled twice adding 1.05 equivalents of Fmoc-protected amino acid, 1 equivalent of *N*,*N*,*N′*,*N′*-tetramethyl-*O*-(1*H*-benzotriazol-1-yl)uranium hexafluorophosphate and 1 equivalent of *N*-methylmorpholine with 5-fold excess over the resin. Peptides were cleaved from the resin and deprotected by incubation of the resin for 3 h with 1 ml of cleavage mixture containing 97.5% trifluoroacetic acid (TFA) and 2.5% water, leaving all peptides amidated at the C-terminus. Peptides were precipitated by the addition of 5 ml of ice-cold diethyl ether and pelleted by centrifugation. The resulting pellets were washed twice with diethyl ether. High-performance liquid chromatography (HPLC) purification was performed on a Gilson Preparative HPLC System using a Zorbax 300SB-C18 column (5 µm particle size, 250 × 9.4 mm) run at 4 ml/min. The solvents used were A (99.9% water and 0.1% formic acid or TFA) and B (94.9% acetonitrile, 5% water and 0.1% formic acid or TFA). A linear gradient from 0% to 10% B was used. All the peptides were retained during the run but were eluted before the gradient, i.e. in 100% A, except for ARTAATARKS and ARTKQTARKS, which were eluted at the beginning of the gradient. Removal of formic acid and TFA was performed using the VAriPure IPE column, and the absence of TFA was confirmed by ^19^F NMR. Purified peptides were submitted to liquid chromatography–mass spectrometry (LC–MS) analysis (Supplementary Figure S13). LC–MS analyses were performed with an Agilent Technologies 1200 series HPLC connected to an Agilent Technologies 6130 quadrupole spectrometer and a diode array detector. Chromatography runs were conducted with a Waters XBridge C18 column, 50 mm × 2.1 mm, with a 3.5 µm particle size with a mobile phase of water/acetonitrile +0.1% formic acid using a gradient from 95:5 to 10:90 over 7.5 min.

### NMR spectroscopy

NMR spectra were acquired from ^15^N or ^15^N/^13^C-labeled samples of BAZ2A PHD and BAZ2B PHD at a concentration of 350 µM in a buffer containing 50 mM KCl, 1 mM dithiothreitol (DTT), 0.02% (w/v) NaN_3_, 10% D_2_O and 25 mM K_2_HPO_4_ at a pH of 6.9 for BAZ2A PHD and a pH of 6.5 for BAZ2B PHD. All NMR experiments were performed at 25°C using an AV-500 MHz Bruker spectrometer equipped with a 5 mm CTPXI ^1^H-^13^C/^15^N/D Z-GRD cryoprobe. Sequence-specific backbone assignments were obtained for BAZ2A PHD and BAZ2B PHD from the identification of intra- and inter-residue resonances in the following spectra: [^15^N-^1^H]-HSQC, ^15^N/^13^C/^1^H HNCO, HNCA, HNCACB and HN(CO)CACB. Acquisition times used in the [^15^N-^1^H]-HSQC experiments were 120 ms for ^1^H and 60 ms for ^15^N. Typical acquisition times in the three-dimensional experiments were: 100 ms for ^1^H, 14–19 ms for ^15^N and 7–12 ms for ^13^C. All the NMR spectra were processed using the program TopSpin (Bruker) and analyzed using the package CcpNmr Analysis [70].

In CSP experiments, the chemical shift differences in proton (Δ*δ*_H_) and nitrogen (Δ*δ*_N_) were combined to obtain a weighted chemical shift difference (Δ*δ*_weighted_) using the following equation: Δ*δ*_weighted_ = |Δ*δ*_H_| + |Δ*δ*_N_| * 0.14, where 0.14 is a scaling factor required to account for the difference in the range of amide proton and amide nitrogen chemical shifts [71]. Shifted residues were clustered based on the extent to which they showed a CSP into strong (Δ*δ*_weighted_ value above the average chemical shift plus two times the standard deviation), medium (Δ*δ*_weighted_ value above the average chemical shift plus the standard deviation) and weak (Δ*δ*_weighted_ value above the average chemical shift). CSPs in the slow exchange regime on the NMR timescale were analyzed using the ‘minimal shift approach’ [72]. The chemical shift change for each backbone amide group was measured from the peak detected in the HSQC spectrum recorded on the free form to the nearest peak detected in the HSQC spectrum recorded on the bound form. Δ*δ*_H_ and Δ*δ*_N_ were combined as described before to obtain a minimal Δ*δ*_weighted_.

### Isothermal titration calorimetry

All calorimetric experiments were performed on a MicroCal iTC_200_ microcalorimeter (GE Healthcare) at 25°C in a buffer containing 20 mM HEPES at a pH of 8, 150 mM NaCl and 0.5 mM Tris(2-carboxyethyl) phosphine. All ITC experiments were carried out titrating peptide solutions (1.5–3 mM) into protein solutions (80–120 µM) loaded in the calorimeter cell, performing one first injection of 0.4 µl followed by 19 injections of 2 µl. The data were analyzed using the MicroCal^™^ software package subtracting the data from an independent titration of peptide into buffer to account for heat of dilution, and then fitted using a single-binding site model. Protein concentration was determined by measuring absorbance at 280 nm using the following extinction coefficients: BAZ2A PHD *ε*_280_ = 6990 M^−1^ cm^−1^ and BAZ2B PHD *ε*_280_ = 8480 M^−1^ cm^−1^. Lyophilized peptides were weighted and dissolved in an appropriate volume of buffer to obtain the desired concentration.

### X-ray crystallography

Crystals of BAZ2A PHD in the *apo* form were grown at 18°C using the sitting drop vapor diffusion method by mixing equal volumes of protein [6 mg/ml in 20 mM Tris–HCl (pH 8), 150 mM NaCl and 2 mM DTT] and crystallization buffer (2.2 M Na/K phosphate buffer at a pH of 8.5). To obtain crystals of the BAZ2A PHD-H3 10-mer complex, preformed *apo* BAZ2A PHD crystals were transferred and soaked overnight into a solution containing 2 mM H3 10-mer (ARTKQTARKS) in crystallization buffer and cryoprotected in 1.6 mM H3 10-mer, 1.7 M Na/K phosphate and 20% glycerol. The data sets were collected at the beamline ID29 at European Synchrotron Radiation Facility and processed with XDS [73,74] and AIMLESS [75], to 2.4 Å of resolution. The structure of the complex was determined by isomorphous replacement with the *apo* form of BAZ2A PHD (PDB entry: 4QF2 [26]). Manual model building and refinement were carried out using Coot [76] and Refmac5 [77]. The quality of the models was checked by MolProbity [78], and all structure figures were generated using PyMOL (The PyMOL Molecular Graphics System, Version 1.7.05, Schrödinger, LLC).

### Molecular dynamics

#### System set-up

The X-ray crystal structure of BAZ2A PHD in complex with H3 10-mer (ARTKQTARKS) was used as the starting structure of the corresponding simulation. The missing residues K9 and S10 were added choosing a suitable low-energy rotamer from PyMOL and minimized for 4000 steps with the rest of the protein fixed. The initial structures of the H3 10-mer with K4A/Q5A and K4G/Q4G mutations to generate ARTAATARKS and ARTGGTARKS, respectively, were built from the WT structure, with the point mutations performed in PyMOL. The structures of the H3 peptides obtained this way were used to simulate the peptides in the unbound state, i.e. in aqueous solution, as well. All models were solvated in a TIP3P water box with a padding of 15 Å from the edge of the box to any protein atom. The system charges were neutralized with sodium or chloride ions as appropriate.

#### Simulation protocol

MD simulations were carried out using the NAMD program [79] and the CHARMM 36 force field [80]. Initially, the solvated systems were minimized for 3000 steps with the protein restrained to eliminate residue unfavorable interactions between the protein and the solvent, followed by another 5000 steps with all atoms free to move. Heating of the systems from 0 to 300 K was achieved in 100 ps (time step of 1 fs), with fixed protein backbone atoms to allow relaxation of the solvent. The systems were subsequently equilibrated for 600 ps (time step of 2 fs) with all atoms free to move. The NPT ensemble was used during the production simulations, which involved four replicates of 80 ns each (time step of 2 fs). The temperature was controlled with a Langevin thermostat at 300 K, and the pressure with a Nose–Hoover Langevin piston barostat at 1 bar. A SHAKE constraint was applied to all bonds containing hydrogen atoms. Short-range non-bonded interactions were switched at 10 Å and cut off at 12 Å, and particle mesh Ewald summation was employed for long-range non-bonded interactions. Consistency and stability throughout the MD replicas were assessed (Supplementary Table S5). The per-residue secondary structure calculation was performed using the Timeline plugin v.2.3 and the hydrogen-bond contacts with the HBonds plugin v.1.2, both contained in VMD v. 1.9.2 [81]. Pair-wise distribution differences among simulated systems were assessed statistically using the two-tailed Mann–Whitney *U*-test, as implemented in the statistical package R [82].

### Sequence alignment

Sequences of domains that belong to the PHD family and whose structures were solved in complex with an H3 N-terminal tail peptide were identified with the software Dali [83] using as input the structure of BAZ2A PHD (4QF2). The sequences of human PHD fingers were obtained from the Structural Genomic Consortium database [1]. The multiple sequence alignment was performed using MAFFT (Multiple Alignment using Fast Fourier Transform) [84] and analyzed using Jalview [85].

### Circular dichroism

CD spectra were acquired from H3-derived peptides dissolved in water (30 µM) at increasing concentrations of TFE using a Bio-Logic CD spectrometer with a cuvette with a path length of 1 mm, at a temperature of 20°C, with a bandwidth of 0.5 nm and a sampling time of 0.5 s. Each spectrum represents the average of three accumulations minus the signal from the blank. Additionally, a constant was added or subtracted to CD spectra so that ellipticity at high wavelengths was 0. Spectra deconvolution was performed using the CONTIN algorithm [86] implemented into DichroWeb [87].

### Accession numbers

The atomic coordinates and structure factors have been deposited in the PDB with the accession number: PDB ID: **5T8R** (BAZ2A PHD in complex with H3 10-mer). NMR assignments for BAZ2A PHD and BAZ2B PHD have been deposited in the BMRB with deposition numbers **26** **754** and **25** **988**, respectively.

## Abbreviations

BAZ: bromodomain adjacent to zinc finger
BMRB: biological magnetic resonance bank
BRD: bromodomain
CD: circular dichroism
CSP: chemical shift perturbation
DPF: double PHD finger
DTT: dithiothreitol
HPLC: high-performance liquid chromatography
HSQC: heteronuclear single-quantum coherence
ITC: isothermal titration calorimetry
LB: lysogeny broth
LC–MS: liquid chromatography–mass spectrometry
MD: molecular dynamics
NoRC: nucleolar remodeling complex
PCR: polymerase chain reaction
PDB: Protein Data Bank
PHD: plant homeodomain
PTM: post-translational modification
rDNA: ribosomal DNA
RMSD: root mean square deviation
SNF2h: sucrose nonfermenting protein 2 homolog
TFA: trifluoroacetic acid
TFE: 2,2,2-trifluoroethanol
WT: wild type

## References

[1] Liu L., Zhen X.T., Denton E., Marsden B.D. and Schapira M. (2012) ChromoHub: a data hub for navigators of chromatin-mediated signalling. Bioinformatics 28, 2205–2206 doi:10.1093/bioinformatics/bts340 22718786 PMC3413389

[2] Wysocka J., Swigut T., Xiao H., Milne T.A., Kwon S.Y., Landry J. et al. (2006) A PHD finger of NURF couples histone H3 lysine 4 trimethylation with chromatin remodelling. Nature 442, 86–90 doi:10.1038/nature04815 16728976

[3] Li H., Ilin S., Wang W., Duncan E.M., Wysocka J., Allis C.D. et al. (2006) Molecular basis for site-specific read-out of histone H3K4me3 by the BPTF PHD finger of NURF. Nature 442, 91–95 doi:10.1038/nature04802 16728978 PMC4690523

[4] Shi X., Hong T., Walter K.L., Ewalt M., Michishita E., Hung T. et al. (2006) ING2 PHD domain links histone H3 lysine 4 methylation to active gene repression. Nature 442, 96–99 doi:10.1038/nature04835 16728974 PMC3089773

[5] Peña P.V., Davrazou F., Shi X., Walter K.L., Verkhusha V.V., Gozani O. et al. (2006) Molecular mechanism of histone H3K4me3 recognition by plant homeodomain of ING2. Nature 442, 100–103 doi:10.1038/nature04814 16728977 PMC3190580

[6] Li Y. and Li H. (2012) Many keys to push: diversifying the ‘readership’ of plant homeodomain fingers. Acta Biochim. Biophys. Sin. 44, 28–39 doi:10.1093/abbs/gmr117 22194011

[7] Musselman C.A. and Kutateladze T.G. (2009) PHD fingers: epigenetic effectors and potential drug targets. Mol. Interv. 9, 314–323 doi:10.1124/mi.9.6.7 20048137 PMC2861807

[8] Musselman C.A. and Kutateladze T.G. (2011) Handpicking epigenetic marks with PHD fingers. Nucleic Acids Res. 39, 9061–9071 doi:10.1093/nar/gkr613 21813457 PMC3241642

[9] Musselman C.A., Lalonde M.-E., Côté J. and Kutateladze T.G. (2012) Perceiving the epigenetic landscape through histone readers. Nat. Struct. Mol. Biol. 19, 1218–1227 doi:10.1038/nsmb.2436 23211769 PMC3645987

[10] Iwase S., Xiang B., Ghosh S., Ren T., Lewis P.W., Cochrane J.C. et al. (2011) ATRX ADD domain links an atypical histone methylation recognition mechanism to human mental-retardation syndrome. Nat. Struct. Mol. Biol. 18, 769–776 doi:10.1038/nsmb.2062 21666679 PMC3130887

[11] Zeng L., Zhang Q., Li S., Plotnikov A.N., Walsh M.J. and Zhou M.-M. (2010) Mechanism and regulation of acetylated histone binding by the tandem PHD finger of DPF3b. Nature 466, 258–262 doi:10.1038/nature09139 20613843 PMC2901902

[12] Taverna S.D., Li H., Ruthenburg A.J., Allis C.D. and Patel D.J. (2007) How chromatin-binding modules interpret histone modifications: lessons from professional pocket pickers. Nat. Struct. Mol. Biol. 14, 1025–1040 doi:10.1038/nsmb1338 17984965 PMC4691843

[13] Zeng L., Yap K.L., Ivanov A.V., Wang X., Mujtaba S., Plotnikova O. et al. (2008) Structural insights into human KAP1 PHD finger–bromodomain and its role in gene silencing. Nat. Struct. Mol. Biol. 15, 626–633 doi:10.1038/nsmb.1416 18488044 PMC3331790

[14] Tsai W.-W., Wang Z., Yiu T.T., Akdemir K.C., Xia W., Winter S. et al. (2010) TRIM24 links a non-canonical histone signature to breast cancer. Nature 468, 927–932 doi:10.1038/nature09542 21164480 PMC3058826

[15] Dreveny I., Deeves S.E., Fulton J., Yue B., Messmer M., Bhattacharya A. et al. (2014) The double PHD finger domain of MOZ/MYST3 induces α-helical structure of the histone H3 tail to facilitate acetylation and methylation sampling and modification. Nucleic Acids Res. 42, 822–835 doi:10.1093/nar/gkt931 24150941 PMC3902925

[16] Li S., Yang Z., Du X., Liu R., Wilkinson A.W., Gozani O. et al. (2016) Structural basis for the unique multivalent readout of unmodified H3 tail by *Arabidopsis* ORC1b BAH-PHD cassette. Structure 24, 486–494 doi:10.1016/j.str.2016.01.004 26876097 PMC4775424

[17] Xie S., Jakoncic J. and Qian C. (2012) UHRF1 double tudor domain and the adjacent PHD finger act together to recognize K9me3-containing histone H3 tail. J. Mol. Biol. 415, 318–328 doi:10.1016/j.jmb.2011.11.012 22100450

[18] Mansfield R.E., Musselman C.A., Kwan A.H., Oliver S.S., Garske A.L., Davrazou F. et al. (2011) Plant homeodomain (PHD) fingers of CHD4 are histone H3-binding modules with preference for unmodified H3K4 and methylated H3K9. J. Biol. Chem. 286, 11779–11791 doi:10.1074/jbc.M110.208207 21278251 PMC3064229

[19] Patel D.J. and Wang Z. (2013) Readout of epigenetic modifications. Annu. Rev. Biochem. 82, 81–118 doi:10.1146/annurev-biochem-072711-165700 23642229 PMC4696766

[20] Andrews F.H., Strahl B.D. and Kutateladze T.G. (2016) Insights into newly discovered marks and readers of epigenetic information. Nat. Chem. Biol. 12, 662–668 doi:10.1038/nchembio.2149 27538025 PMC5116920

[21] Eberharter A., Vetter I., Ferreira R. and Becker P.B. (2004) ACF1 improves the effectiveness of nucleosome mobilization by ISWI through PHD-histone contacts. EMBO J. 23, 4029–4039 doi:10.1038/sj.emboj.7600382 15457208 PMC524333

[22] Racki L.R., Yang J.G., Naber N., Partensky P.D., Acevedo A., Purcell T.J. et al. (2009) The chromatin remodeller ACF acts as a dimeric motor to space nucleosomes. Nature 462, 1016–1021 doi:10.1038/nature08621 20033039 PMC2869534

[23] Bochar D.A., Savard J., Wang W., Lafleur D.W., Moore P., Côté J. et al. (2000) A family of chromatin remodeling factors related to Williams syndrome transcription factor. Proc. Natl Acad. Sci. U.S.A. 97, 1038–1043 doi:10.1073/pnas.97.3.1038 10655480 PMC15513

[24] Pascual J., Martinez-Yamout M., Dyson H.J. and Wright P.E. (2000) Structure of the PHD zinc finger from human Williams–Beuren syndrome transcription factor. J. Mol. Biol. 304, 723–729 doi:10.1006/jmbi.2000.4308 11124022

[25] Guetg C., Lienemann P., Sirri V., Grummt I., Hernandez-Verdun D., Hottiger M.O. et al. (2010) The NoRC complex mediates the heterochromatin formation and stability of silent rRNA genes and centromeric repeats. EMBO J. 29, 2135–2146 doi:10.1038/emboj.2010.17 20168299 PMC2905252

[26] Tallant C., Valentini E., Fedorov O., Overvoorde L., Ferguson F.M., Filippakopoulos P. et al. (2015) Molecular basis of histone tail recognition by human TIP5 PHD finger and bromodomain of the chromatin remodeling complex NoRC. Structure 23, 80–92 doi:10.1016/j.str.2014.10.017 25533489 PMC4291147

[27] Santoro R., Li J. and Grummt I. (2002) The nucleolar remodeling complex NoRC mediates heterochromatin formation and silencing of ribosomal gene transcription. Nat. Genet. 32, 393–396 doi:10.1038/ng1010 12368916

[28] Zhou Y. and Grummt I. (2005) The PHD finger/bromodomain of NoRC interacts with acetylated histone H4K16 and is sufficient for rDNA silencing. Curr. Biol. 15, 1434–1438 doi:10.1016/j.cub.2005.06.057 16085498

[29] Gu L., Frommel S.C., Oakes C.C., Simon R., Grupp K., Gerig C.Y. et al. (2015) BAZ2A (TIP5) is involved in epigenetic alterations in prostate cancer and its overexpression predicts disease recurrence. Nat. Genet. 47, 22–30 doi:10.1038/ng.3165 25485837

[30] Jones M.H., Hamana N., Nezu J.-i. and Shimane M. (2000) A novel family of bromodomain genes. Genomics 63, 40–45 doi:10.1006/geno.1999.6071 10662543

[31] Ferguson F.M., Dias D.M., Rodrigues J.P.G.L.M., Wienk H., Boelens R., Bonvin A.M.J.J. et al. (2014) Binding hotspots of BAZ2B bromodomain: histone interaction revealed by solution NMR driven docking. Biochemistry 53, 6706–6716 doi:10.1021/bi500909d 25266743 PMC4458377

[32] Hol W.G.J., van Duijnen P.T. and Berendsen H.J.C. (1978) The α-helix dipole and the properties of proteins. Nature 273, 443–446 doi:10.1038/273443a0 661956

[33] Plotnikov A.N., Yang S., Zhou T.J., Rusinova E., Frasca A. and Zhou M.-M. (2014) Structural insights into acetylated-histone H4 recognition by the bromodomain-PHD finger module of human transcriptional coactivator CBP. Structure 22, 353–360 doi:10.1016/j.str.2013.10.021 24361270 PMC3923519

[34] Rack J.G.M., Lutter T., Kjæreng Bjerga G.E., Guder C., Ehrhardt C., Värv S. et al. (2014) The PHD finger of p300 influences its ability to acetylate histone and non-histone targets. J. Mol. Biol. 426, 3960–3972 doi:10.1016/j.jmb.2014.08.011 25158095

[35] Klein B.J., Piao L., Xi Y., Rincón-Arano H., Rothbart S.B., Peng D. et al. (2014) The histone-H3K4-specific demethylase KDM5B binds to its substrate and product through distinct PHD fingers. Cell Rep. 6, 325–335 doi:10.1016/j.celrep.2013.12.021 24412361 PMC3918441

[36] Torres I.O., Kuchenbecker K.M., Nnadi C.I., Fletterick R.J., Kelly M.J.S. and Fujimori D.G. (2015) Histone demethylase KDM5A is regulated by its reader domain through a positive-feedback mechanism. Nat. Commun. 6, 6204 doi:10.1038/ncomms7204 25686748 PMC5080983

[37] Chakravarty S., Essel F., Lin T. and Zeigler S. (2015) Histone peptide recognition by KDM5B-PHD1: a case study. Biochemistry 54, 5766–5780 doi:10.1021/acs.biochem.5b00617 26266342

[38] Chen Y., Wan B., Wang K.C., Cao F., Yang Y., Protacio A. et al. (2011) Crystal structure of the N-terminal region of human Ash2L shows a winged-helix motif involved in DNA binding. EMBO Rep. 12, 797–803 doi:10.1038/embor.2011.101 21660059 PMC3147254

[39] Ali M., Hom R.A., Blakeslee W., Ikenouye L. and Kutateladze T.G. (2014) Diverse functions of PHD fingers of the MLL/KMT2 subfamily. Biochim. Biophys. Acta, Mol. Cell Res. 1843, 366–371 doi:10.1016/j.bbamcr.2013.11.016 PMC3925188 24291127

[40] Klein B.J., Wang X., Cui G., Yuan C., Botuyan M.V., Lin K. et al. (2016) PHF20 readers link methylation of histone H3K4 and p53 with H4K16 acetylation. Cell Rep. 17, 1158–1170 doi:10.1016/j.celrep.2016.09.056 27760318 PMC5125728

[41] Pace C.N. and Scholtz J.M. (1998) A helix propensity scale based on experimental studies of peptides and proteins. Biophys. J. 75, 422–427 doi:10.1016/S0006-3495(98)77529-0 9649402 PMC1299714

[42] Greenfield N.J. (2007) Using circular dichroism spectra to estimate protein secondary structure. Nat. Protoc. 1, 2876–2890 doi:10.1038/nprot.2006.202 PMC2728378 17406547

[43] Nelson J.W. and Kallenbach N.R. (1986) Stabilization of the ribonuclease S-peptide α-helix by trifluoroethanol. Proteins 1, 211–217 doi:10.1002/prot.340010303 3449856

[44] Jasanoff A. and Fersht A.R. (1994) Quantitative determination of helical propensities from trifluoroethanol titration curves. Biochemistry 33, 2129–2135 doi:10.1021/bi00174a020 8117669

[45] Luo P. and Baldwin R.L. (1997) Mechanism of helix induction by trifluoroethanol: a framework for extrapolating the helix-forming properties of peptides from trifluoroethanol/water mixtures back to water. Biochemistry 36, 8413–8421 doi:10.1021/bi9707133 9204889

[46] Matsubara M., Hayashi N., Titani K. and Taniguchi H. (1997) Circular dichroism and 1H NMR studies on the structures of peptides derived from the calmodulin-binding domains of inducible and endothelial nitric-oxide synthase in solution and in complex with calmodulin. Nascent α-helical structures are stabilized by calmodulin both in the presence and absence of Ca^2+^. J. Biol. Chem. 272, 23050–23056 doi:10.1074/jbc.272.37.23050 9287303

[47] Zhang Y., Yang H., Guo X., Rong N., Song Y., Xu Y. et al. (2014) The PHD1 finger of KDM5B recognizes unmodified H3K4 during the demethylation of histone H3K4me2/3 by KDM5B. Protein Cell 5, 837–850 doi:10.1007/s13238-014-0078-4 24952722 PMC4225485

[48] Wang C., Shen J., Yang Z., Chen P., Zhao B., Hu W. et al. (2011) Structural basis for site-specific reading of unmodified R2 of histone H3 tail by UHRF1 PHD finger. Cell Res. 21, 1379–1382 doi:10.1038/cr.2011.123 21808299 PMC3193461

[49] Arita K., Isogai S., Oda T., Unoki M., Sugita K., Sekiyama N. et al. (2012) Recognition of modification status on a histone H3 tail by linked histone reader modules of the epigenetic regulator UHRF1. Proc. Natl Acad. Sci. U.S.A. 109, 12950–12955 doi:10.1073/pnas.1203701109 22837395 PMC3420164

[50] Cheng J., Yang Y., Fang J., Xiao J., Zhu T., Chen F. et al. (2013) Structural insight into coordinated recognition of trimethylated histone H3 lysine 9 (H3K9me3) by the plant homeodomain (PHD) and tandem tudor domain (TTD) of UHRF1 (ubiquitin-like, containing PHD and RING finger domains, 1) protein. J. Biol. Chem. 288, 1329–1339 doi:10.1074/jbc.M112.415398 23161542 PMC3543016

[51] Hu L., Li Z., Wang P., Lin Y. and Xu Y. (2011) Crystal structure of PHD domain of UHRF1 and insights into recognition of unmodified histone H3 arginine residue 2. Cell Res. 21, 1374–1378 doi:10.1038/cr.2011.124 21808300 PMC3193472

[52] Rajakumara E., Wang Z., Ma H., Hu L., Chen H., Lin Y. et al. (2011) PHD finger recognition of unmodified histone H3R2 links UHRF1 to regulation of euchromatic gene expression. Mol. Cell 43, 275–284 doi:10.1016/j.molcel.2011.07.006 21777816 PMC4691841

[53] Lallous N., Legrand P., McEwen A.G., Ramón-Maiques S., Samama J.-P. and Birck C. (2011) The PHD finger of human UHRF1 reveals a new subgroup of unmethylated histone H3 tail readers. PLoS ONE 6, e27599 doi:10.1371/journal.pone.0027599 22096602 PMC3214078

[54] Qiu Y., Liu L., Zhao C., Han C., Li F., Zhang J. et al. (2012) Combinatorial readout of unmodified H3R2 and acetylated H3K14 by the tandem PHD finger of MOZ reveals a regulatory mechanism for HOXA9 transcription. Genes Dev. 26, 1376–1391 doi:10.1101/gad.188359.112 22713874 PMC3387664

[55] Li W., Zhao A., Tempel W., Loppnau P. and Liu Y. (2016) Crystal structure of DPF3b in complex with an acetylated histone peptide. J. Struct. Biol. 195, 365–372 doi:10.1016/j.jsb.2016.07.001 27402533

[56] Xiong X., Panchenko T., Yang S., Zhao S., Yan P., Zhang W. et al. (2016) Selective recognition of histone crotonylation by double PHD fingers of MOZ and DPF2. Nat. Chem. Biol. 12, 1111–1118 doi:10.1038/nchembio.2218 27775714 PMC5253430

[57] Klein B.J., Simithy J., Wang X., Ahn J.W., Andrews F.H., Zhang Y. et al. (2017) Recognition of histone H3K14 acylation by MORF. Structure doi:10.1016/j.str.2017.02.003 PMC5415407 28286003

[58] Wang X., Moore S.C., Laszckzak M. and Ausio J. (2000) Acetylation increases the α-helical content of the histone tails of the nucleosome. J. Biol. Chem. 275, 35013–35020 doi:10.1074/jbc.M004998200 10938086

[59] Zhang G., Smith S.G. and Zhou M.-M. (2015) Discovery of chemical inhibitors of human bromodomains. Chem. Rev. 115, 11625–11668 doi:10.1021/acs.chemrev.5b00205 26492937

[60] Galdeano C. and Ciulli A. (2016) Selectivity on-target of bromodomain chemical probes by structure-guided medicinal chemistry and chemical biology. Fut. Med. Chem. 8, 1655–1680 doi:10.4155/fmc-2016-0059 PMC5321501 27193077

[61] Herold J.M., Wigle T.J., Norris J.L., Lam R., Korboukh V.K., Gao C. et al. (2011) Small-molecule ligands of methyl-lysine binding proteins. J. Med. Chem. 54, 2504–2511 doi:10.1021/jm200045v 21417280 PMC3109722

[62] Wagner T., Robaa D., Sippl W. and Jung M. (2014) Mind the methyl: methyllysine binding proteins in epigenetic regulation. ChemMedChem. 9, 466–483 doi:10.1002/cmdc.201300422 24449612

[63] Milosevich N. and Hof F. (2016) Chemical inhibitors of epigenetic methyllysine reader proteins. Biochemistry 55, 1570–1583 doi:10.1021/acs.biochem.5b01073 26650180

[64] Simhadri C., Daze K.D., Douglas S.F., Quon T.T.H., Dev A., Gignac M.C. et al. (2014) Chromodomain antagonists that target the polycomb-group methyllysine reader protein chromobox homolog 7 (CBX7). J. Med. Chem. 57, 2874–2883 doi:10.1021/jm401487x 24625057

[65] Ren C., Morohashi K., Plotnikov A.N., Jakoncic J., Smith S.G., Li J. et al. (2015) Small-molecule modulators of methyl-lysine binding for the CBX7 chromodomain. Chem. Biol. 22, 161–168 doi:10.1016/j.chembiol.2014.11.021 25660273 PMC4336573

[66] Stuckey J.I., Dickson B.M., Cheng N., Liu Y., Norris J.L., Cholensky S.H. et al. (2016) A cellular chemical probe targeting the chromodomains of Polycomb repressive complex 1. Nat. Chem. Biol. 12, 180–187 doi:10.1038/nchembio.2007 26807715 PMC4755828

[67] Miller T.C.R., Rutherford T.J., Birchall K., Chugh J., Fiedler M. and Bienz M. (2014) Competitive binding of a benzimidazole to the histone-binding pocket of the Pygo PHD finger. ACS Chem. Biol. 9, 2864–2874 doi:10.1021/cb500585s 25323450 PMC4330097

[68] Wagner E.K., Nath N., Flemming R., Feltenberger J.B. and Denu J.M. (2012) Identification and characterization of small molecule inhibitors of a plant homeodomain finger. Biochemistry 51, 8293–8306 doi:10.1021/bi3009278 22994852 PMC3567257

[69] Marley J., Lu M. and Bracken C. (2001) A method for efficient isotopic labeling of recombinant proteins. J. Biomol. 20, 71–75 doi:10.1023/A:1011254402785 11430757

[70] Vranken W.F., Boucher W., Stevens T.J., Fogh R.H., Pajon A., Llinas M. et al. (2005) The CCPN data model for NMR spectroscopy: development of a software pipeline. Proteins 59, 687–696 doi:10.1002/prot.20449 15815974

[71] Williamson M.P. (2013) Using chemical shift perturbation to characterise ligand binding. Prog. Nucl. Mag. Res. Spectr. 73, 1–16 doi:10.1016/j.pnmrs.2013.02.001 23962882

[72] Farmer B.T., Constantine K.L., Goldfarb V., Friedrichs M.S., Wittekind M., Yanchunas J. et al. (1996) Localizing the NADP^+^ binding site on the MurB enzyme by NMR. Nat. Struct. Biol. 3, 995–997 doi:10.1038/nsb1296-995 8946851

[73] Kabsch W. (2010) *XDS* Acta Crystallogr. D Biol. Crystallogr. 66, 125–132 doi:10.1107/S0907444909047337 20124692 PMC2815665

[74] Kabsch W. (2010) Integration, scaling, space-group assignment and post-refinement. Acta Crystallogr. D Biol. Crystallogr. 66, 133–144 doi:10.1107/S0907444909047374 20124693 PMC2815666

[75] Evans P.R. and Murshudov G.N. (2013) How good are my data and what is the resolution? Acta Crystallogr. D Biol. Crystallogr. 69, 1204–1214 doi:10.1107/S0907444913000061 23793146 PMC3689523

[76] Emsley P., Lohkamp B., Scott W.G. and Cowtan K. (2010) Features and development of *Coot*. Acta Crystallogr. D Biol. Crystallogr. 66, 486–501 doi:10.1107/S0907444910007493 20383002 PMC2852313

[77] Murshudov G.N., Vagin A.A. and Dodson E.J. (1997) Refinement of macromolecular structures by the maximum-likelihood method. Acta Crystallogr. D Biol. Crystallogr. 53, 240–255 doi:10.1107/S0907444996012255 15299926

[78] Chen V.B., Arendall W.B., Headd J.J., Keedy D.A., Immormino R.M., Kapral G.J. et al. (2010) *Molprobity*: all-atom structure validation for macromolecular crystallography. Acta Crystallogr. D Biol. Crystallogr. 66, 12–21 doi:10.1107/S0907444909042073 20057044 PMC2803126

[79] Phillips J.C., Braun R., Wang W., Gumbart J., Tajkhorshid E., Villa E. et al. (2005) Scalable molecular dynamics with NAMD. J. Comput. Chem. 26, 1781–1802 doi:10.1002/jcc.20289 16222654 PMC2486339

[80] Brooks B.R., Brooks C.L., Mackerell A.D., Nilsson L., Petrella R.J., Roux B. et al. (2009) CHARMM: the biomolecular simulation program. J. Comput. Chem. 30, 1545–1614 doi:10.1002/jcc.21287 19444816 PMC2810661

[81] Humphrey W., Dalke A. and Schulten K. (1996) VMD: visual molecular dynamics. J. Mol. Graphics 14, 33–38 doi:10.1016/0263-7855(96)00018-5 8744570

[82] R Development Core Team (2013) R: A language and environment for statistical computing, Vienna, Austria

[83] Holm L. and Rosenström P. (2010) Dali server: conservation mapping in 3D. Nucleic Acids Res. 38, W545–W549 doi:10.1093/nar/gkq366 20457744 PMC2896194

[84] Katoh K. and Standley D.M. (2013) MAFFT multiple sequence alignment software version 7: improvements in performance and usability. Mol. Biol. Evol. 30, 772–780 doi:10.1093/molbev/mst010 23329690 PMC3603318

[85] Waterhouse A.M., Procter J.B., Martin D.M.A., Clamp M. and Barton G.J. (2009) Jalview Version 2—a multiple sequence alignment editor and analysis workbench. Bioinformatics 25, 1189–1191 doi:10.1093/bioinformatics/btp033 19151095 PMC2672624

[86] Provencher S.W. and Glöckner J. (1981) Estimation of globular protein secondary structure from circular dichroism. Biochemistry 20, 33–37 doi:10.1021/bi00504a006 7470476

[87] Whitmore L. and Wallace B.A. (2008) Protein secondary structure analyses from circular dichroism spectroscopy: methods and reference databases. Biopolymers 89, 392–400 doi:10.1002/bip.20853 17896349

